# Depression and executive functioning bidirectionally impair one another across 9 years: Evidence from within-person latent change and cross-lagged models

**DOI:** 10.1192/j.eurpsy.2021.2217

**Published:** 2021-06-17

**Authors:** Nur Hani Zainal, Michelle G. Newman

**Affiliations:** 1 National University of Singapore, Kent Ridge Campus, Singapore; 2 The Pennsylvania State University, State College, Pennsylvania, USA

**Keywords:** anxiety, depression, executive functioning, latent change, random-intercept cross-lagged panel models

## Abstract

**Background:**

Scar and vulnerability models assert that increased psychopathology may predict subsequent executive functioning (EF) deficits (and vice versa) over protracted timescales, yet most prior work on this topic has been cross-sectional. Thus, we tested the *within-* and between-person relations between EF, depression, and anxiety.

**Methods:**

Older adult participants (*n* = 856) were assessed across four waves, approximately 2 years apart. Performance-based EF and caregiver-rated symptom measures were administered. Bivariate latent change score and random-intercept cross-lagged panel models were conducted.

**Results:**

Within persons, random-intercept cross-lagged panel models revealed that prior greater depression forecasted lower subsequent EF, and vice versa (*d* = −0.292 vs. −0.292). Bivariate dual latent change score models showed that within-person rise in depression predicted EF decreases, and vice versa (*d* = −0.245 vs. −0.245). No within-person, cross-lagged, EF-anxiety relations emerged. Further, significant negative between-person EF-symptom relations were observed (*d* = −0.264 to −0.395).

**Conclusion:**

Prospective, within-person findings offer some evidence for developmental scar and vulnerability models.

In daily life, most of us depend on our global executive functioning (EF) capacity to effectively accomplish tasks, communicate, handle emotions, make choices, prioritize goals, and solve problems [1,2]. Global EF is defined as a group of multidomain cognitive control systems entwined with attention, information processing, and other cognitive abilities [3,4]. Our global EF systems comprise facets of inhibition (capacity to abstain from autopilot actions), working memory (WM; ability to alter cognitive representations with incoming data in real-time), shifting (adeptness to flexibly switch from one mental set to another) [5], and verbal fluency [6]. Relatedly, evidence has shown consistently that language-based, temporal lobe-mediated verbal fluency ability (marked by scores on diverse time-limited word generation on animal- and phonemic-cued tests) had strong and unique relations with common EF variance (i.e., global EF capacity) in diverse youth and adult samples [7–10]. Given its importance, EF problems have been linked to issues with career, social relationships, diet, nutrition, and health [11,12]. Executive dysfunction-related health problems include cardiorespiratory, metabolic, neuroendocrine, and psychiatric disorders [13,14]. Thus, understanding the risk factors and consequences of EF decrements is essential.

*Scar theories* propose that increases in psychiatric symptoms can precede and predict future EF decline. Specifically, scar models posit that chronic increased depression and anxiety may build up oxidative and inflammatory-stress, thereby adversely impacting EF-related brain regions over protracted durations [15–17]. Relatedly, scar models such as the vascular- [18] and executive dysfunction syndrome-depression [19] hypotheses assert that increased depression and anxiety could impair future EF via buildup of tissue injury (e.g., lacunes, microinfarcts, and white matter hyperintensities) in cardiovascular systems, cognitive control-, and reward processing-related brain regions, over long timescales [20]. These brain areas might include frontal–striatal pathways (e.g., dorsolateral prefrontal cortex, basal ganglia, thalamus, and anterior cingulate cortex) [21,22].

Thus far, 47 longitudinal studies have offered support for scar models. For instance, higher depression severity during adolescence was associated with lower vocabulary score in early adulthood 8 years later [23]; however, whether such pattern applied to various stages in adulthood could not be inferred from that study. Other studies suggested such a possibility. Swedish and American adults with (vs. without) major depression displayed worsened episodic memory, EF, or verbal fluency after 6 months to 5 years despite symptom remission [24,25]. Likewise, among mid-life and older community adults, increased anxiety was related to reduced immediate and delayed auditory memory abilities following 12 years [26]. Similarly, 2 meta-analyses of 43 studies showed that heightened anxiety and depression dovetailed with larger EF decline and incidence of major neurocognitive disorders in diverse community and clinical samples across 1–17 years [27,28].

Simultaneously, *vulnerability models* argue that EF decline can function as a precursor of later heightened depression and anxiety. Vulnerability models assert that poorer EF may forecast future anxiety and depression across prolonged periods due to chronic problems with disengaging from negative self-referential perseverative thinking (e.g., worry and rumination) [29,30]. Likewise, EF deficits can make it perpetually hard to detach from threats, leading to excessive focus on anxiety-inducing factors in one’s surroundings and risk for increased anxiety [31,32]. Moreover, it has been thought that poorer EF, especially WM, can predict increased depression and anxiety across long durations, in part due to difficulties with adjusting to various changing emotion-eliciting contexts in versatile and optimal ways [33]. In sum, vulnerability theories argue that worse EF may forecast increased depression and anxiety over long durations.

To date, 31 prospective investigations have empirically supported vulnerability theories. For example, an earlier study demonstrated that poorer WM was related to future chronic course of increased depression [34]. Likewise, reduced inhibition, WM, shifting, verbal fluency, and other cognitive functioning indices were connected with pathological worry dimensionally and categorically 9 years later in community adults [35]. More recently, meta-analytic data on 29 studies (*n* = 121,749) showed that cognitive deficits were associated with increased major depression severity following several months to 45 years in diverse clinical and community-dwelling samples [36].

However, the mostly two-time-point, between-person, regression studies testing the prospective relations between mental health symptoms and global EF to date introduce shortcomings to clinical science. Such methods do not account for the nesting of repeated assessments within persons to capture change-to-future change trajectories across time [37]. Mounting global pressures related to neuropsychiatric illnesses, increasing life expectancy, and aging [38–40] make it crucial to explore whether change in global EF over long durations may be related to future change in mental illness during adulthood development. Further, between-person differences across time may be due to stable variations observed across the lifespan [41], or to individual differences in aging-associated rate of EF decrements [42–45]. The latter possibility can only be captured by using within-person methods that also capture change. Moreover, the foregoing scar and vulnerability theories posit that EF-symptom relations unfold within persons across long durations [46–51]. Awareness of within-person prolonged trajectories of increased depression or anxiety, EF decrements, and their covariation may guide the design of personalized prevention, diagnostic, and treatment efforts that rely on idiographic (or within-person) more than between-person data, as part of precision psychiatry [52–54]. It is also important to note that observations of between- and within-person differences in EF and psychopathological symptoms do not always align with each other [55–57]. To broaden and deepen comprehension of EF and mental health in mid-life and older adulthood, within-person (co)variations and change must be considered. Tethering within-person data analytic approaches with longitudinal study designs is thus important to comprehend the bidirectional within-person changes in EF and subsequent changes in symptoms (and vice versa).

Two cutting-edge techniques that attain these aims are random-intercept cross-lagged panel models (RI-CLPM) [58] and bivariate dual latent change score models (BLCS) [59]; two forms of longitudinal structural equation modeling (SEM). These longitudinal SEM approaches benefit researchers by accounting for prior lagged relations and regression to the mean, minimizing measurement unreliability, and using all available values instead of listwise deletion [57]. Further, by adjusting for temporally stable between-person differences and autoregressive effects, these models can test if change in one variable across a previous time-period or time-lag is associated with change in another variable at the next time-period or time-lag within persons. Accordingly, by evaluating lead–lag change-to-future change connections, RI-CLPM and BLCS models move us toward the ability to draw causal inferences [60]; inquiries essential to clinical science.

Thus far, three studies of adult participants have tested the longitudinal, dynamic, within-person relations between EF and anxiety, depression, or pertinent concepts with BLCS. Using BLCS, increase in anxiety was related to cognitive functioning decline in older adults [57]; despite that, the two-wave study prevented understanding of how symptom change predicted subsequent EF change (and conversely). Relatedly, BLCS models across three waves showed that 9-year growth in excessive worry dovetailed with future 9-year decline in global and unique EF facets [49]; however, whether change in EF forecasted later change in worry was not examined. Another five time-point study demonstrated that rise in trait neuroticism at one time-lag preceded and linked to reductions in spatial processing, WM, and processing speed at the next time-lag [57]; nonetheless, one-item assessments of cognitive functioning were used in the study. To our knowledge, no studies have tested EF-psychological symptom relations in older adults with RI-CLPM. However, a recent study in youths that utilized RI-CLPM [61] suggested the possibility of EF problems serving as risk factors for later increased depression and anxiety.

Building on this literature, this study aimed to examine the *within-*person associations between a global EF composite (formed via a latent composite of five measures) and depression or anxiety severity using RI-CLPM and BLCS in older adults. Based on scar theories, we hypothesized that within persons, higher anxiety or depression severity would reliably precede and relate to greater future EF decline at the next time-point and time-lag. Moreover, based on vulnerability models, we hypothesized that within persons, lower EF would forecast subsequent rise or increased depression or anxiety severity at the next time-point or time-lag. Last, using a SEM-based model comparison approach [62], we aimed to directly juxtapose the effect sizes indicating the scar (vs. vulnerability) hypothesis to determine if any differences emerged.

## Method

### Participants

The present study was a secondary analysis of the Aging, Demographics, and Memory Study (ADAMS) publicly available and restricted-use datasets [63]. Ethical approval was provided by the University of Michigan and Duke University Medical Center, and all participants voluntarily consented to enroll. Participants (*n* = 856) averaged 81.59 years of age (*SD* = 7.10, range = 70–110), 58.53% were female, and 76.87% identified as White, compared to the other 23.13% who identified African American or other ethnicities. In addition, 28.62% (*n* = 245) of the participants needed support for dressing, feeding, or bathing based on caregiver report, or were diagnosed with Diagnostic and Statistical Manual-Fourth Edition-Text Revised (DSM-IV-TR) [64,65]—defined dementia, major depressive disorder, stroke, or other neurological condition. These dementia syndromes included probable and possible Alzheimer’s disease, cardiovascular, and other causes. All DSM-IV-TR diagnoses were attained through expert consensus with a multidisciplinary team of neurologists, psychiatrists, geriatric psychologists, and other healthcare professionals [66]. The online supplementary material (OSM) offers more details on the sample characteristics.

### Procedures

Participants completed performance-based EF measures and had a significant other caregiver (e.g., spouse and children) who could reliably report on their behavioral symptoms across multiple time-points. Data were collected across four waves in 2004 (Time 1; T1), 2006 (Time 2; T2), 2008 (Time 3; T3), and 2010 (Time 4; T4) [66]. The following caregiver-rated symptom assessment and EF tests were administered.

### Measures

#### Mental health symptoms

The widely used caregiver-rated Neuropsychiatric Inventory (NPI) [67]—depression and anxiety domains were utilized to assess past-month depression and anxiety severity in the form of a structured interview. Caregiver ratings were used in this study as self-reported mental health symptom severity data was only available at T1 and T2 [63,68,69], and due to the reliable nature of caregiver-reported data that tends to align with self-rated symptom measures [70]. To measure depression, caregivers were asked about the presence and duration of depression symptoms (e.g., sadness, irritability, feels worthless, and suicidal thoughts) the participant may have exhibited. To assess for anxiety, caregivers were inquired on the presence of any anxiety symptoms (e.g., excessive worry, breathlessness, and behavioral avoidance). Also, for each participant’s symptom domain, the informants reported on the degree of the following four facets: severity (3-point scale; 1 = *mild* to 3 = *marked*); change from past typical behaviors (3-point scale; 0 = *no*; 1 = *yes*; 2 = *exaggeration of previous problems*); distress (6-point scale; 0 = *not at all* to 5 = *very severely or extremely*). Supplementary Tables S1 and S2 in the OSM show that these four manifest indicators for the depression and anxiety scales had excellent model fit using a series of confirmatory factor analysis (CFA) at distinct time-points. Further, the NPI has reliably shown strong internal consistency, as well as convergent and discriminant validity [67,71]. In this study, internal consistencies were high for the depression severity (Cronbach’s αs = 0.93, 0.94, 0.92, and 0.93 at T1, T2, T3, and T4, respectively) and anxiety severity scales (αs = 0.93, 0.96, 0.93, and 0.90 at T1, T2, T3, and T4, respectively).

#### Executive functioning

The following five measures of EF were used to create a composite latent global EF composite: (a) controlled oral word association (a verbal fluency assessment that captures unplanned generation of words within a time limit that start with some assigned letter) [72]; (b) animal fluency (another time-limited verbal fluency test based on the animal category) [73]; (c) serial 7 subtraction (extent of accuracy of counting down from 100 by 7 within a time limit) [74]; (d) backward digit span (degree of accuracy of recall in reverse order of integer strings of increasing length) [75]; and (e) symbol digit modality test (level of accuracy of replacing a single-digit integer for randomized displays of geometric patterns) [76]. These EF assessments have been shown to have good internal consistency, strong convergent, and discriminant validity [77–79]. In this study, the αs for the global EF composite were strong across all time-points (αs = 0.92, 0.83, 0.87, and 0.88 at T1, T2, T3, and T4, respectively). Moreover, at each time-point, a composite global EF index was created by standardizing each EF measure and averaging the scores across EF measures. Further, exploratory factor analysis and a series of CFA demonstrated that a one-factor latent global EF composite had good model fit across waves of assessment (refer to page 4 and Supplementary Table S3 of the OSM). In addition, these global EF scores have been normed based on age and education, and appropriate adjustments were made for participants with hearing impairments [80–83]. Also, Supplementary Table S4 in the OSM shows the descriptive statistics of the study variables based on SEM analyses.

### Data analyses

All longitudinal SEM analyses were performed with the *lavaan* package [84] in *R* Version 3.6.3. Model fit was assessed utilizing practical fit indices and heuristic cut-offs: confirmatory fit index (CFI; CFI ≥ 0.90) [85] and root mean square error of approximation (RMSEA; RMSEA ≤ 0.09) [86]. To maximize all available data points, we used full information maximum likelihood, the gold standard [87], to manage missing data. In total, 16.29% of the data were missing. Further, the data were missing completely at random (χ^2^[*df* = 113] = 134.32, *p* = 0.084).

Next, we established longitudinal measurement invariance; a prerequisite for longitudinal SEM [88]. We progressively evaluated for *configural* invariance (equivalence of factor structure), *metric* invariance (equal factor structure and item loadings [λs], freely estimated item intercepts [τs], and item error variances [εs] across the time-points), *scalar* invariance (equal factor structure, λs, and τs, but freely estimated εs across time-points), and *strict* invariance (equal factor structure, λs, τs, and εs, across time-points) [62]. To test for measurement invariance, we conducted a Δχ^2^ difference test. A statistically significant Δχ^2^ meant that the more (vs. less) restricted model had poorer fit [89]. However, as Δχ^2^ is affected by sample size despite negligible misfit changes, the following change in practical fit indices, ΔCFI ≤ −0.01 or ΔRMSEA < +0.015 [90], from the less restricted to more restricted models signaled measurement nonequivalence.

The RI-CLPM was used to manage interdependent repeated assessments nested within persons, and to distinguish between within-person (dynamic state) variance and between-person (trait) variance [91]. RI-CLPM procedures permitted us to test these within-person reciprocal cross-lagged relations (γs) accounting for within-person autoregressive effects (βs; level of one variable forecasting its subsequent level), trait variances (αs), and regression to the mean [92,93]. Of primary interest were the within-person cross-lagged associations between *level* of EF at a prior time-point (T − 1) and *level* of depression or anxiety symptom severity (SYM) at the next adjacent time-point (T) following about 2 years (γs) (and vice versa), as shown in Equations (1) and (2).(1)
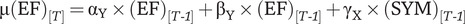

(2)
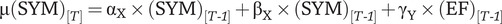
Concurrently, BLCS approaches were utilized to test if within-person change in depression or anxiety symptom severity at a previous time-lag (ΔT − 1) would be related to *change* in EF at the next successive time-lag (ΔT) (and vice versa). BLCS is a cutting-edge method that empowers researchers to test within-person change-to-future change associations (*coupling effects*; δs) after accounting for trait-level initial status, trait-level *constant change* parameters (αs), and within-person autoregressive paths (*proportional effects*; change in a variable predicting subsequent change in itself; βs) [59]. The BLCS models relevant to our research question can be denoted in Equations (3) and (4) as follows.(3)
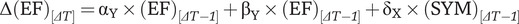

(4)
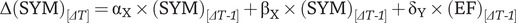
As recommended, the within-person cross-lagged associations (γs) in the RI-CLPM and within-person coupling effects in the BLCS (δs) were constrained to be equal across waves of assessments to reduce *SEs* in parameter estimates (refer to Supplementary Figure S1 shows a BLCS model in [94]). Also, baseline psychopathology and EF were controlled for in all models.

As we aimed to directly compare the scar and vulnerability hypotheses, we contrasted a model that freely estimated the cross-lagged or coupling effects (EF predicting future SYM and conversely) to a model that constrained the cross-lagged or coupling effects to equality. A statistically significant change (Δ) in χ^2^ value in comparing these two models would indicate notable differences in the strength of effect sizes for one hypothesis versus the other [62]. If the Δχ^2^ test was not significant, the more parsimonious model with equality constraints on cross-lagged or coupling effects was chosen as the final model. Effect sizes were calculated using the formula, Cohen’s 
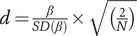
 [95], where β is the unstandardized regression estimate, *SD*(β) its standard deviation, and *N* is the sample size. Cohen’s *d* values of 0.2, 0.5, and 0.8 indicated small, moderate, and large effects, respectively.

### Power analysis

Following best practices [96], an *a priori* Monte Carlo power analysis based on a conservative effect size of *d* = 0.20 for the cross-lagged effects in the RI-CLPM and coupling effects (bidirectional change-to-future change EF-symptom relations) in the BLCS was performed using the RAMpath *R* package [97]. After 1,000 replications per condition, we observed 90.6–100.0% power to detect significant within-person cross-lagged or coupling effects. Further, there was 90.0–100.0% power to identify other significant parameter estimates.

## Results

### Longitudinal measurement invariance

Supplementary Table S5 in the OSM shows the longitudinal measurement invariance analyses for the constructs of interest. Overall, *strict* level of equivalence (equal λs, τs, εs) was observed for the constructs of depression severity, anxiety severity, and EF. Therefore, conducting analyses using RI-CLPM and BLCS approaches were appropriate.

### Lagged relations between depression severity and executive function

Table 1 displays all of the parameter estimates for the RI-CLPM testing the cross-lagged relations between depression severity and EF. The model with equality constraints on the cross-lagged effects did not significantly differ from the model that freely estimated those parameters (Δχ^2^[*df* = 1] = 0.017, *p* = 0.895). The parsimonious model with equality constraints showed good model fit (χ^2^[*df* = 24] = 45.160, *p* = 0.006, CFI = 0.984, RMSEA = 0.032). Within persons, higher prior depression severity substantially predicted lower EF at the subsequent time-point (β = −0.073, 95% CI [−0.119, −0.026], *d* = −0.292). Likewise, lower previous EF significantly predicted greater depression severity at the next time-point within persons (β = −0.073, 95% CI [−0.119, −0.026], *d* = −0.292). Also, between persons, higher random intercept depression severity was significantly correlated with lower random intercept EF (β = −0.055, 95% CI [−0.094, −0.016], *d* = −0.264).

**Table 1.** Random-intercepts cross-lagged panel model of DEP and EF across four time-points.

	Estimate	95% CI	Cohen’s *d*
Within-person cross-lagged effects			
(DEP)_[*T-1*]_ ➔ (EF)_[*T*]_	−0.073**	[−0.119, −0.026]	−0.292
(EF)_[*T-1*]_ ➔ (DEP)_[*T*]_	−0.073**	[−0.119, −0.026]	−0.292
Within-person autoregressive effects			
(DEP)_[*T-1*]_ ➔ (DEP)_[*T*]_	0.248***	[0.157, 0.338]	0.518
(EF)_[*T-1*]_ ➔ (EF)_[*T*]_	0.289**	[0.074, 0.504]	0.252
Between-person covariances			
(EF)_[*T*]_ ↔ (DEP)_[*T*]_	−0.027***	[−0.042, −0.011]	−0.324
RI(EF)_[*T*]_ ↔ RI(DEP)_[*T*]_	−0.055**	[−0.094, −0.016]	−0.264
RI(DEP)_[*T*]_ ↔ (DEP)_[*T1*]_	0.006	[−0.067, 0.078]	0.016
RI(EF)_[*T*]_ ↔ (EF)_[*T1*]_	−0.121***	[−0.176, −0.066]	−0.415
Between-person intercepts			
Mean of (DEP)_[*T1*]_	0.371***	[0.316, 0.426]	1.272
Mean of (DEP)_[*T2*]_	0.256***	[0.176, 0.336]	0.599
Mean of (DEP)_[*T3*]_	0.290***	[0.212, 0.368]	0.696
Mean of (DEP)_[*T4*]_	0.266***	[0.173, 0.360]	0.532
Mean of (EF)_[*T1*]_	−0.000	[−0.058, 0.058]	0.000
Mean of (EF)_[*T2*]_	−0.094*	[−0.168, −0.020]	−0.237
Mean of (EF)_[*T3*]_	−0.655***	[−0.735, −0.575]	−1.534
Mean of (EF)_[*T4*]_	−0.785***	[−0.872, −0.698]	−1.713
Residual variances			
Variance of RI(DEP)_[*T1*-T4]_	0.000	–	–
Variance of RI(EF)_[*T1*-*T4*]_	0.904***	[0.783, 1.025]	1.400
Variance of (DEP)_[*T1*]_	0.670***	[0.513, 0.827]	0.804
Variance of (EF)_[*T1*]_	0.089***	[0.059, 0.120]	0.534
Variance of (DEP)_[*T2*-*T4*]_	0.412***	[0.371, 0.453]	1.883
Variance of (EF)_[*T2*-*T4*]_	0.088***	[0.067, 0.109]	0.768

*Note:* Model fit indices: χ^2^(*df* = 24) = 45.160, *p* = 0.006, CFI = 0.984, RMSEA = 0.032, 95% CI [0.017, 0.046]. Within-person cross-lagged effects refer to level in DEP at a prior time-point (T-1) predicting (➔) future Δ in EF at the next adjacent time-point (T) (and vice versa). Within-person coupling effects and proportional effects, residual covariances between DEP and EF, as well as variances of DEP and EF were each uniquely fixed to be equal across all three time-lags.

Abbreviations: CI, confidence interval; DEP, depression severity; EF, executive functioning; RI, random intercept.

* *p* < 0.05.

** *p* < 0.01.

*** *p* < 0.001.

**Figure 1.**
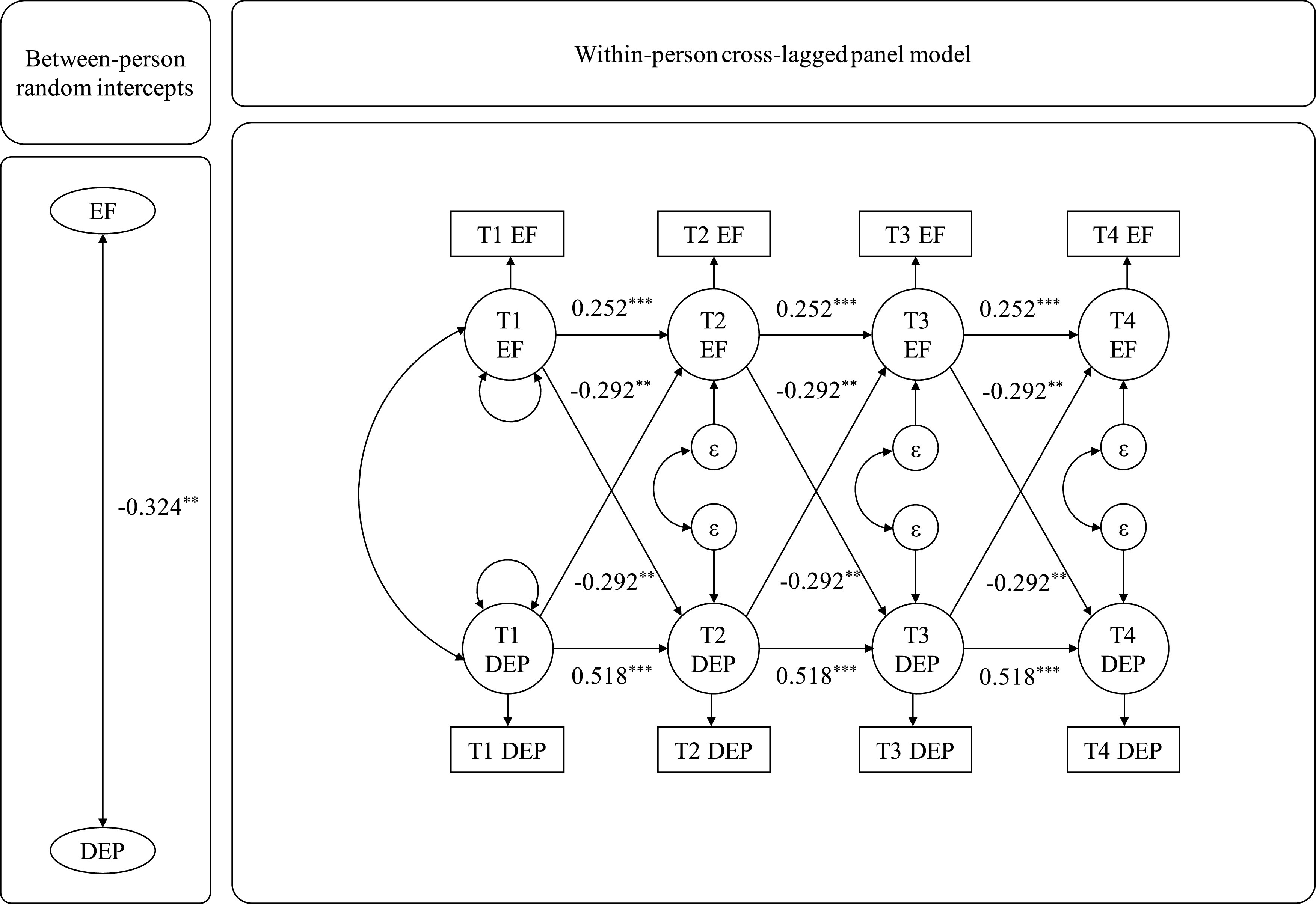
Random-Intercept Cross-Lagged Panel Models Between EF and Depression Severity. *Note*. **p < .01; ***p < .001. Δ = within-person change in construct from a time-lag to the next adjacent time-lag; DEP = depression severity; EF = executive functioning.

**Figure 2.**
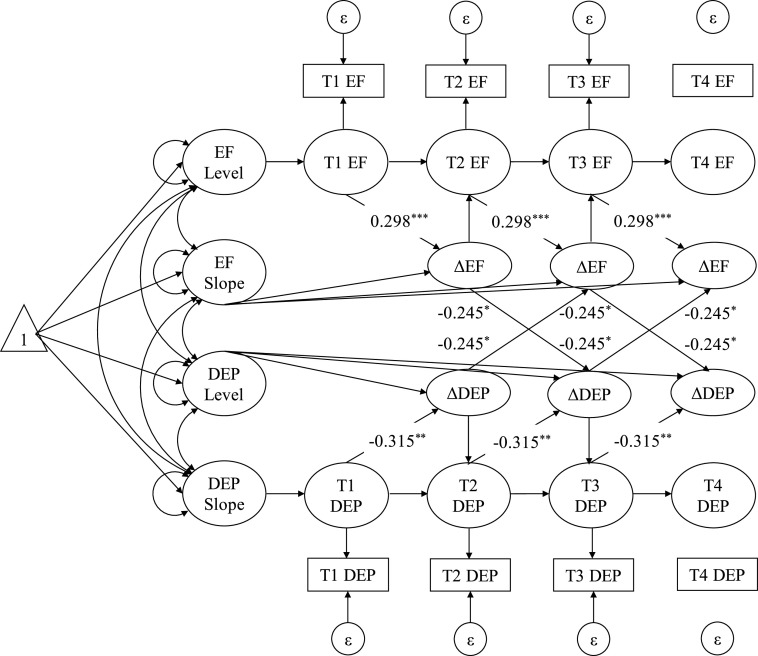
Bivariate Dual Latent Change Score Models Between EF and Depression Severity. *Note*. **p < .01; ***p < .001. Δ = within-person change in construct from a time-lag to the next adjacent time-lag; DEP = depression severity; EF = executive functioning.

Table 2 presents the parameter estimates for the BLCS models examining the change-to-future change associations between depression severity and EF. The freely estimated (vs. constrained) models were not significantly different from each other (Δχ^2^[*df* = 1] = 0.235, *p* = 0.628). The final model with equality constraints on the coupling effects showed acceptable model fit (χ^2^[*df* = 25] = 47.000, *p* = 0.005, CFI = 0.974, RMSEA = 0.039, 95% CI [0.021, 0.057]). Within persons, greater growth in depression severity at a prior time-lag significantly predicted EF decrement at the next time-lag (β = −0.540, 95% CI [−0.955, −0.124], *d* = −0.245). Likewise, within persons, EF decline at a previous time-lag was significantly associated with larger increase in depression severity at the subsequent time-lag (β = −0.540, 95% CI [−0.955, −0.124], *d* = −0.245). Figures 1 and 2 summarize the analyses of the lagged relations between depression severity and EF.

**Table 2.** Bivariate dual latent change score model of DEP and EF across four time-points.

	Estimate	95% CI	Cohen’s *d*
Within-person coupling effects			
(DEP)_[*ΔT-1*]_ ➔ Δ(EF)_[*ΔT*]_	−0.540*	[−0.955, −0.124]	−0.245
(EF)_[*ΔT-1*]_ ➔ Δ(DEP)_[*ΔT*]_	−0.540*	[−0.955, −0.124]	−0.245
Within-person proportional effects			
(DEP)_[*ΔT-1*]_ ➔ Δ(DEP)_[*ΔT*]_	−0.462***	[−0.738, −0.185]	0.315
(EF)_[*ΔT-1*]_ ➔ Δ(EF)_[*ΔT*]_	0.093**	[0.033, 0.152]	−0.298
Between-person covariances			
(DEP)_[*T-1*]_ ↔ Δ(DEP)_[*ΔT*]_	0.010	[−0.038, 0.059]	0.038
(EF)_[*T-1*]_ ↔ Δ(EF)_[*ΔT*]_	−0.021	[−0.058, −0.017]	−0.106
(EF)_[*T-1*]_ ↔ Δ(DEP)_[*T-1*]_	−0.092***	[−0.139, −0.045]	−0.368
(EF)_[*ΔT*]_ ↔ Δ(DEP)_[*ΔT*]_	−0.010*	[−0.018, 0.001]	−0.192
(DEP)_[*T-1*]_ ↔ Δ(EF)_[*ΔT*]_	−0.014	[−0.032, 0.005]	−0.149
(EF)_[*T-1*]_ ↔ Δ(DEP)_[*ΔT*]_	0.002	[−0.031, 0.035]	−0.011
(EF)_[*T*]_ ↔ Δ(DEP)_[*T*]_	0.003	[−0.016, 0.022]	−0.029
Between-person intercepts			
Mean of (DEP)_[*T1*]_	0.367***	[0.313, 0.422]	1.258
Mean of Δ(DEP)_[*T*]_	0.079	[−0.000, 0.159]	0.185
Mean of (EF)_[*T1*]_	0.003	[−0.055, −0.061]	0.010
Mean of Δ(EF)_[*T*]_	−0.103***	[−0.137, −0.068]	−0.549
Variances			
Variance of (DEP)_[*T1*]_	0.278***	[0.152, 0.405]	0.411
Residuals of Δ(DEP)_[*T*]_	0.395***	[0.315, 0.475]	0.925
Variance of Δ(DEP)_[*T*]_	0.000	–	–
Variance of (EF)_[*T*1]_	0.696***	[0.646, 0.746]	2.673
Residuals of Δ(EF)_[*T*]_	0.067***	[0.055, 0.079]	1.072
Variance of Δ(EF)_[*T*]_	0.000	–	–

*Note:* Model fit indices: *χ* ^2^(*df* = 25) = 47.000, *p* = 0.005, CFI = 0.974, RMSEA = 0.039, 95% CI [0.021, 0.057]. Within-person coupling effects refer to change (Δ) in DEP at a prior time-lag (ΔT-1) predicting (➔) future Δ in EF at the next adjacent time-lag (ΔT; and vice versa). Within-person coupling effects and proportional effects, residual covariances between DEP and EF, as well as variances of DEP and EF were each uniquely fixed to be equal across all three time-lags.

Abbreviations: CI, confidence interval; DEP, depression severity; EF, executive functioning.

* *p* < 0.05;

** *p* < 0.01;

*** *p* < 0.001.

### Lagged relations between anxiety severity and executive function

Table 3 shows the model parameter estimates for the RI-CLPM evaluating the cross-lagged relations between anxiety severity and EF. The freely estimated model was not significantly different from the constrained model (Δχ^2^[*df* = 1] = 0.069, *p* = 0.792). The final model with equality constraints on the cross-lagged effects demonstrated good model fit (χ^2^[*df* = 23] = 86.84, *p* < 0.001, CFI = 0.952, RMSEA = 0.057). Within persons, no cross-lagged relations were observed between prior anxiety severity and EF at the subsequent time-point (β = −0.025, 95% CI [−0.101, 0.051], *d* = −0.062). Likewise, no within-person cross-lagged relations were found between previous EF and anxiety severity at the next time-point (β = −0.025, 95% CI [−0.101, 0.051], *d* = −0.062). However, between persons, higher random intercept anxiety severity was significantly related to lower random intercept EF (β = −0.070, 95% CI [−0.104, −0.036], *d* = −0.395).

**Table 3.** Random-intercepts cross-lagged panel model of ANX and EF across four time-points.

	Estimate	95% CI	Cohen’s *d*
Within-person cross-lagged effects			
(ANX)_[*T-1*]_ ➔ (EF)_[*T*]_	−0.025	[−0.101, 0.051]	−0.062
(EF)_[*T-1*]_ ➔ (ANX)_[*T*]_	−0.025	[−0.101, 0.051]	−0.062
Within-person autoregressive effects			
(ANX)_[*T-1*]_ ➔ (ANX)_[*T*]_	0.120*	[0.026, 0.213]	0.245
(EF)_[*T-1*]_ ➔ (EF)_[*T*]_	0.287	[0.070, 0.643]	0.151
Between-person covariances			
(EF)_[*T*]_ ↔ (ANX)_[*T*]_	−0.008	[−0.020, 0.003]	−0.128
RI(EF)_[*T*]_ ↔ RI(ANX)_[*T*]_	−0.070***	[−0.104, −0.036]	−0.395
RI(ANX)_[*T*]_ ↔ (ANX)_[*T1*]_	0.104*	[0.006, 0.201]	0.200
RI(EF)_[*T*]_ ↔ (EF)_[*T1*]_	−0.125***	[−0.175, −0.074]	−0.462
Between-person intercepts			
Mean of (ANX)_[*T1*]_	0.229***	[0.182, 0.277]	0.916
Mean of (ANX)_[*T2*]_	0.141***	[0.064, 0.217]	0.347
Mean of (ANX)_[*T3*]_	0.176***	[0.109, 0.243]	0.497
Mean of (ANX)_[*T4*]_	0.149***	[0.76, 0.222]	0.387
Mean of (EF)_[*T1*]_	−0.000	[−0.058, 0.058]	0.000
Mean of (EF)_[*T2*]_	−0.096*	[−0.171, −0.021]	−0.243
Mean of (EF)_[*T3*]_	−0.657***	[−0.733, −0.582]	−1.617
Mean of (EF)_[*T4*]_	−0.789***	[−0.875, −0.704]	−1.762
Variances			
Variance of RI(ANX)_[*T1*-*T4*]_	0.000	–	–
Variance of RI(EF)_[*T1*-*T4*]_	0.912***	[0.816, 1.009]	1.786
Variance of (ANX)_[*T1*]_	0.297**	[0.107, 0.486]	1.584
Variance of (EF)_[*T1*]_	0.088***	[0.052, 0.123]	0.338
Variance of (ANX)_[*T2*-*T4*]_	0.262***	[0.151, 0.373]	0.441
Variance of (EF)_[*T2*-*T4*]_	0.090***	[0.058, 0.122]	0.540

*Note:* Model fit indices: χ^2^(*df* = 24) = 33.102, *p* = 0.102, CFI = 0.982, RMSEA = 0.021, 95% CI [0.008, 0.031]. Within-person cross-lagged effects refer to level in ANX at a prior time-point (T-1) predicting (➔) future Δ in EF at the next adjacent time-point (T) (and vice versa). Within-person coupling effects and proportional effects, residual covariances between ANX and EF, as well as variances of ANX and EF were each uniquely fixed to be equal across all three time-lags.

Abbreviations: ANX, anxiety severity; CI, confidence interval; EF, executive functioning; RI, random intercept.

* *p* < 0.05.

** *p* < 0.01.

*** *p* < 0.001.

Table 4 shows the parameter estimates for the BLCS models testing the change-to-future change relations between anxiety severity and EF. The constrained (vs. freely estimated) models were not significantly different (Δχ^2^[*df* = 1] = 0.005, *p* = 0.943). The final model with equality constraints on the coupling parameters showed acceptable model fit (χ^2^[*df* = 25] = 46.996, *p* < 0.001, CFI = 0.966, RMSEA = 0.057). Within persons, prior change in anxiety severity at a previous time-lag was not significantly associated with change in EF at the subsequent time-lag (β = −0.254, 95% CI [−0.951, 0.444], *d* = −0.068) and vice versa (β = −0.254, 95% CI [−0.951, 0.444], *d* = −0.068).^1^

**Table 4.** Bivariate dual latent change score model of ANX and EF across four time-points.

	Estimate	95% CI	Cohen’s *d*
Within-person coupling effects			
(ANX)_*[ΔT-1*]_ ➔ Δ(EF)_[*ΔT*]_	−0.254	[−0.951, 0.444]	−0.068
(EF)_[*ΔT-1*]_ ➔ Δ(ANX)_[*ΔT*]_	−0.254	[−0.951, 0.444]	−0.068
Within-person proportional effects			
(ANX)_[*ΔT-1*]_ ➔ Δ(ANX)_[*ΔT*]_	0.006	[−0.597, 0.608]	0.002
(EF)_[*ΔT-1*]_ ➔ Δ(EF)_[*ΔT*]_	0.080**	[0.025, 0.135]	0.274
Between-person covariances			
(ANX)_[*T-1*]_ ↔ Δ(ANX)_[*ΔT*]_	−0.049***	[−0.077, −0.021]	−0.336
(EF)_[*T-1*]_ ↔ Δ(EF)_[*ΔT*]_	−0.014	[−0.044, 0.016]	−0.090
(EF)_[*T-1*]_ ↔ Δ(ANX)_[*T-1*]_	−0.101***	[−0.144, −0.058]	−0.441
(EF)_[*ΔT*]_ ↔ Δ(ANX)_[*ΔT*]_	−0.007	[−0.017, 0.002]	−0.134
(ANX)_[*T-1*]_ ↔ Δ(EF)_[*ΔT*]_	0.007	[−0.016, 0.030]	0.056
(EF)_[*T-1*]_ ↔ Δ(ANX)_[*ΔT*]_	0.031	[−0.016, 0.078]	0.124
(EF)_[*T*]_ ↔ Δ(ANX)_[*T*]_	0.004	[−0.009, 0.018]	0.055
Between-person intercepts			
Mean of (ANX)_[*T1*]_	0.224***	[0.178, 0.270]	0.935
Mean of Δ(ANX)_[*T*]_	−0.052	[−0.155, 0.051]	−0.096
Mean of (EF)_[*T1*]_	0.003	[−0.055, 0.061]	0.010
Mean of Δ(EF)_[*T*]_	−0.102***	[−0.136, −0.068]	−0.576
Variances			
Variance of (ANX)_[*T1*]_	0.231*	[0.056, 0.407]	0.246
Residuals of Δ(ANX)_[*T*]_	0.256***	[0.138, 0.374]	0.410
Variance of Δ(ANX)_[*T*]_	0.000	–	–
Variance of (EF)_[*T1*]_	0.692***	[0.642, 0.742]	2.657
Residuals of Δ(EF)_[*T*]_	0.071***	[0.058, 0.084]	0.974
Variance of Δ(EF)_[*T*]_	0.000	–	–

*Note:* Model fit indices: χ^2^(*df* = 25) = 46.996, *p* < 0.001, CFI = 0.966, RMSEA = 0.057, 95% CI [0.044, 0.071]. Within-person coupling effects refer to change (Δ) in ANX at a prior time-lag (ΔT-1) predicting (➔) future Δ in EF at the next adjacent time-lag (ΔT; and vice versa). Within-person coupling effects and proportional effects, residual covariances between ANX and EF, as well as variances of ANX and EF were each uniquely fixed to be equal across all three time-lags.

Abbreviations: ANX, anxiety severity; CI, confidence interval; EF, executive functioning.

* *p* < 0.05.

** *p* < 0.01.

*** *p* < 0.001.

## Discussion

Partially supporting scar and vulnerability hypotheses, robust RI-CLPM and BLCS methods showed that within persons, higher prior level and change in depression (but not anxiety) severity predicted greater reduced EF at the next time-point and subsequent time-lag, and conversely. Simultaneously, these models demonstrated stronger between-person, cross-sectional magnitude between EF and anxiety compared to EF and depression severity. Overall, findings concurred with up-to-date, cross-sectional, between-person evidence from recent meta-analytic data (e.g., [36]). Results also extended an early seminal cross-sectional study [98] which observed that whereas patients with (vs. without) depression performed poorly on auditory and visual WM tasks, patients with anxiety disorders attained scores comparable to healthy controls. Findings also built on hierarchical linear modeling results that whereas inverse EF-depression relations tended to predominate within persons, negative EF-anxiety associations tended to be larger between persons [99]. The divergence between within- and between-person findings for anxiety is likely due to the fact that between-person analyses do not account for individual differences in person-specific changes across time. Whereas between-person differences across time could be due to group differences in stable variations observed across the lifespan, they may not be capturing individual differences in aging-associated rate of EF or mental health deterioration. In fact, whereas prior between-person findings were interpreted to suggest that moderate levels of anxiety (but not depression) could facilitate performance on EF tests up to a certain point, this relation has not held up when examined at the within-person level [100–102]. Another potential explanation pertains to the fact that anxiety (vs. depression) severity tends to be more stable across the lifespan, as illustrated by prospective [103] and gene–environment studies [104]. Accordingly, higher stability and lower variability in anxiety severity across the lifespan could translate to stronger predominance of between-person, as opposed to within-person, effects on EF over long durations. Clearly, more longitudinal work is needed to test these notions.

Why did rise in depression severity consistently predict future EF decline at the next time-point and time-lag within persons? Overall, our findings offered support for scar theories. Conceivably, recurrent depression episodes might be a factor in cognitive functioning decline and diseased neurological aging processes (e.g., shrinkage in learning- and EF-linked brain regions and white matter hyperintensities) over the years [105,106]. Biologically, elevated depression might have this adverse effect on EF across prolonged durations via chronic wear-and-tear of the hypothalamic–pituitary–adrenal axis function, such as buildup of glucocorticoids and proinflammatory cytokines (e.g., C-reactive protein) [107,108]. On that note, elevated depression might precede or speed up the onset of dementia, possibly via the accumulation of neurofibrillary plaques and tangles in emotion modulation-, EF-, and learning-related brain areas [109–111]. Equally tenable are scar models centering on behavioral, environmental, and lifestyle factors observed for extended durations in depression (e.g., decreased physical exercise, suboptimal sleep, diet, and nutrition), that could impact proinflammatory and cardiovascular processes [112,113]. Future longitudinal studies using RI-CLPM and BLCS models can further examine the “neurotoxic” scar effect of increased depression.

Findings suggested that reduced EF functioned as a risk factor for subsequent heightened depression (but not anxiety) within persons. This could be because poorer EF may have compromised abilities to harness “top-down” cognitive control over depressed mood (but not necessarily anxiety symptoms), and to refocus thoughts and actions to create and sustain more positive emotions (e.g., via engaging in mood-lifting activities or searching for suitable social support). However, the result that change in EF deficits did not forecast change in future anxiety within persons was inconsistent with prior longitudinal, between-person studies that found evidence supporting the vulnerability hypothesis. For instance, two studies showed that EF deficits were risk factors for generalized anxiety disorder symptoms across time (e.g., [35,114]). Also, using BLCS, two studies observed that within-person rise in anxiety or trait neuroticism at a time-lag predicted worsened cognitive functioning at the next time-lag [57,115] in community-dwelling Swedish adults. Similarly, another recent BLCS study found that within persons, worse cognitive functioning forecasted increased anxiety and depression across 4 years in patients with Parkinson’s disease [116]. Differences in data analysis (e.g., linear regression vs. SEM), sample characteristics (e.g., age and data collection site), anxiety measures (e.g., worry vs. anxiety symptoms), and study design (e.g., time-lags) might account for such variability in findings.

In addition, *between* persons, *cross-sectional* relations between lower EF and greater depression or anxiety severity were reliably observed. Observations at the between-person level are concordant with several community-based studies. For example, poorer EF facets (e.g., shifting and inhibition) or global cognition have been shown to consistently forecast increased worry, anxiety, and depression at a later time in children [117], adolescents [118], mid-life adults [35], and older adults [114], across 3–12 years. Our study extended those findings by bolstering arguments that the strength and sign of magnitudes between within- and between-person associations might not coincide [56]. The field can benefit from using prospective designs (e.g., cross-panel and ecological momentary assessment) and SEM to clarify the between- and within-person relations among EF, depression, and anxiety severity across years and smaller timescales (e.g., within-day and day-to-day fluctuations) [48,119].

Relatedly, the *cross-sectional, between-person* negative associations between anxiety or depression and EF in this study may be accounted for by the *attentional control theory* [120] and *attentional scope model of rumination* [121]. Note that these theories are inappropriate for explaining the within-person, cross-lagged, and long-term change-to-future change relations between EF and depression severity found herein as they argue that symptom-EF perturbation relations occur across brief durations or at one time-point [49,122]. Further, these models assert that elevated symptoms could deplete finite EF resources for task-pertinent processing and increased anxiety and depression would be reliably linked to greater cognitive rigidity (i.e., difficulty disengaging from threat or distractions) at a single time-point. Such mechanisms may unfold through excessive repetitive negative thinking, such as worry, brooding, and obsessions, as consistently evidenced by cross-sectional or experimental meta-analytic data [123–125].

Findings must be interpreted in light of study strengths and limitations. Unmeasured third variables (e.g., genetics) [126] may have contributed to observed outcomes. Additionally, although other studies have observed within- or between-person relations between depression and EF domains of shifting and inhibition [49], consistent with theory and neuroanatomical evidence [127], these EF facets were not measured herein. Also, as no structured diagnostic interviews were included, future studies that include such diagnostic instruments could determine if the results would be similar. In addition, given the predominantly White sample, subsequent investigations can determine if outcomes extend to culturally diverse populations by conducting multiple-group SEM (e.g., [128]). Limitations notwithstanding, study strengths included the large and well-powered sample size, administration of behavioral EF and caregiver-rated symptom assessments, four-wave cross-panel longitudinal dataset, and use of potent SEM approaches.

If the pattern of results herein was replicated, some clinical implications deserve consideration. Offering preventive interventions at early signals of increased depression might assist with remediating depression, but would also probably benefit EF capacities. Moreover, the field could benefit from continuing to test EF indices as reliable predictors or markers of treatment response for depression and anxiety, as suggested by various studies (e.g., [129,130]). Relatedly, based on recent evidence, such efforts might be augmented by investigating if cognitive-behavioral therapies (CBTs) (e.g., behavioral activation, cognitive remediation, problem-solving therapy, personalized, environment-focused, and technology-facilitated CBTs) [131–136], mindfulness-based interventions [137], EF training [138], and pharmacological treatments [139], could simultaneously alleviate depression and enhance EF capacities.

## Data Availability

The data that support the findings of this study are available from Health and Retirement Study (HRS)—ADAMS—website. Data are available at https://hrs.isr.umich.edu/publications/biblio/5761 with the permission of the study team principal investigators and team members, G. H. Steven, G. F. Gwenith, D. H. Michael, M. L. Kenneth, O. Mary Beth, L. P. Brenda, R. W. David, and colleagues.

## Footnotes

1 As part of a sensitivity analysis, we determined that the results were similar when analyses were restricted to a sample without baseline major depression and cognitive or physical disabilities (*n* = 611), as shown on page 9 of the OSM.

## Supplementary material

For supplementary material accompanying this paper visit http://dx.doi.org/10.1192/j.eurpsy.2021.2217. click here to view supplementary material
