# Supplementary material for: Depression and executive functioning bidirectionally impair one another across 9 years: Evidence from within-person latent change and cross-lagged models
Source: Eur Psychiatry. 2021 Jun 17;64(1):e43. doi: 10.1192/j.eurpsy.2021.2217 (PMC8278253; doi:10.1192/j.eurpsy.2021.2217)
Supplement: Supplementary file 1 [file S0924933821022173sup001.docx]

**Supplementary Materials**

## Sample Characteristics

Specifically, the proportion of participants with a current major depressive episode (i.e., past 2-week period of depression, loss of interest, and irritability, with problems related to appetite, sleep, restlessness, fatigue, worthlessness/guilt, concentration, suicidality) was 13.32% (*n* = 114). In addition, based on self-reported psychiatric diagnoses, 0.35% (*n* = 3) of participants had lifetime bipolar disorder, 0.70% (*n* = 6) had lifetime schizophrenia, 0.82% (*n* = 7) had intellectual disability, and 0.12% (*n* = 1) had current or lifetime alcohol use disorder. In addition, some of these participants were diagnosed by physicians with medical conditions such as coronary obstructive pulmonary disease, congestive heart failure, chronic pain, delirium, and other medical problems, and had other neurological conditions (*n* = 68). Whereas 89.14% (*n* = 763) were community dwelling adults, 10.86% (*n* = 93) were nursing home residents.

Table S1

*Confirmatory factor analysis of latent depression composite scores at all time-points*

|  |  | Factor loadings |  | Residual variances |
| --- | --- | --- | --- | --- |
| **Time 1** |  |  |  |  |
| Model fit indices: χ^2^(*df* = 2) = 0.295, *p = .*863, CFI = 1.000, RMSEA = .000 |  |  |  |  |
| Presence of symptom |  | 0.912^***^ |  | 0.168^***^ |
| Severity |  | 0.967^***^ |  | 0.065^***^ |
| Change from past typical behaviors |  | 0.781^***^ |  | 0.391^***^ |
| Distress |  | 0.829^***^ |  | 0.313^***^ |
| Latent symptom composite |  | – |  | 1.000^***^ |
| **Time 2** |  |  |  |  |
| Model fit indices: χ^2^(*df* = 2) = 0.386, *p = .*825, CFI = 1.000, RMSEA = .000 |  |  |  |  |
| Presence of symptom |  | 0.888^***^ |  | 0.212^***^ |
| Severity |  | 0.960^***^ |  | 0.079^***^ |
| Change from past typical behaviors |  | 0.864^***^ |  | 0.254^***^ |
| Distress |  | 0.815^***^ |  | 0.336^***^ |
| Latent symptom composite |  | – |  | 1.000^***^ |
| **Time 3** |  |  |  |  |
| Model fit indices: χ^2^(*df* = 2) = 0.065, *p = .*968, CFI = 1.000, RMSEA = .000 |  |  |  |  |
| Presence of symptom |  | 0.839^***^ |  | 0.296^***^ |
| Severity |  | 0.979^***^ |  | 0.042^***^ |
| Change from past typical behaviors |  | 0.855^***^ |  | 0.268^***^ |
| Distress |  | 0.759^***^ |  | 0.423^***^ |
| Latent symptom composite |  | – |  | 1.000^***^ |
| **Time 4** |  |  |  |  |
| Model fit indices: χ^2^(*df* = 2) = 0.125, *p = .*940, CFI = 1.000, RMSEA = .000 |  |  |  |  |
| Presence of symptom |  | 0.920^***^ |  | 0.153^***^ |
| Severity |  | 0.907^***^ |  | 0.177^***^ |
| Change from past typical behaviors |  | 0.907^***^ |  | 0.169^***^ |
| Distress |  | 0.792^***^ |  | 0.373^***^ |
| Latent symptom composite |  | – |  | 1.000^***^ |
|  |  |  |  |  |

Table S2

*Confirmatory factor analysis of latent anxiety composite scores at all time-points*

|  |  | Factor loadings |  | Residual variances |
| --- | --- | --- | --- | --- |
| **Time 1** |  |  |  |  |
| Model fit indices: χ^2^(*df* = 2) = 0.196, *p = .*906, CFI = 1.000, RMSEA = .000 |  |  |  |  |
| Presence of symptom |  | 0.924^***^ |  | 0.147^***^ |
| Severity |  | 0.971^***^ |  | 0.057^***^ |
| Change from past typical behaviors |  | 0.833^***^ |  | 0.306^***^ |
| Distress |  | 0.773^***^ |  | 0.403^***^ |
| Latent symptom composite |  | – |  | 1.000^***^ |
| **Time 2** |  |  |  |  |
| Model fit indices: χ^2^(*df* = 3) = 0.013, *p* = 1.000, CFI = 1.000, RMSEA = .000 |  |  |  |  |
| Presence of symptom |  | 0.987^***^ |  | 0.026 |
| Severity |  | 0.953^***^ |  | 0.092^***^ |
| Change from past typical behaviors |  | 0.898^***^ |  | 0.194^***^ |
| Distress |  | 0.882^***^ |  | 0.223^***^ |
| Latent symptom composite |  | – |  | 1.000^***^ |
| **Time 3** |  |  |  |  |
| Model fit indices: χ^2^(*df* = 2) = 0.083, *p = .*959, CFI = 1.000, RMSEA = .000 |  |  |  |  |
| Presence of symptom |  | 0.899^***^ |  | 0.191^***^ |
| Severity |  | 0.958^***^ |  | 0.082^***^ |
| Change from past typical behaviors |  | 0.881^***^ |  | 0.224^***^ |
| Distress |  | 0.793^***^ |  | 0.371^***^ |
| Latent symptom composite |  | – |  | 1.000^***^ |
| **Time 4** |  |  |  |  |
| Model fit indices: χ^2^(*df* = 2) = 0.333, *p = .*847, CFI = 1.000, RMSEA = .000 |  |  |  |  |
| Presence of symptom |  | 0.848^***^ |  | 0.280^***^ |
| Severity |  | 0.922^***^ |  | 0.150^***^ |
| Change from past typical behaviors |  | 0.750^***^ |  | 0.437^***^ |
| Distress |  | 0.744^***^ |  | 0.446^***^ |
| Latent symptom composite |  | – |  | 1.000^***^ |

Factor Analysis for Executive Functioning Constructs

To ascertain the optimal number of factors for the EF items at all time-points, we divided the sample in half (*n* = 428 each) (Matsunaga, 2010; Rosellini & Brown, 2021), and conducted factor analyses in the following sequence, following best practice recommendations: (1) principal components analysis (PCA); (2) exploratory factor analysis (EFA); (3) confirmatory factor analysis (CFA). Whereas PCA and EFA was performed on Sample 1, CFA was conducted on Sample 2. The *R* *psych* package (Revelle, 2020) was used to conduct PCA and EFA on all five EF items: controlled oral word association (COWAT); animal fluency (AF); serial 7 subtraction (S7); backward digit span (BDS); symbol digit modality test (SDMT).

Exploratory Factor Analysis

PCA utilized Promax rotation that allowed EF items to correlate freely considering associations among EF items. Using the .5/.2 cutoff rule, we retained items with factor loadings ≥ .50 on one component and ≤ .20 on the rest of the components. Following this, we performed parallel analysis (PA), a conservative and stringent item selection method that guided the discrimination of unique components (Lim & Jahng, 2019). A random normally distributed dataset with identical sample size and number of variables as the reduced EF item pool was produced. Afterwards, this simulated dataset underwent factor analyses 1,000 times where eigenvalues were calculated via a Jocobi routine (Watkins, 2005). Moreover, average eigenvalues and standard deviations (SDs) were computed across replications and compared with eigenvalues of factors extracted from the original dataset. Only factors that surpassed the average of the parallel factor eigenvalues were retained.

Next, to determine the optimal number of factors to extract to account for the data’s variance and to remove any existing items that did not load on any of the extracted factors, EFA with Promax rotation was conducted on the reduced item pool jointly with PA. PA is a consistent, optimal, and recommended method to extract factors for continuous variables (Lubbe, 2019). Following determination of the optimal number of factors to extract, we performed another EFA, limited to the number of factors. Likewise, the .5/.2 cutoff rule was used to retain EF items.

PCA of five EF items in the ADAMS dataset yielded one major component (item loadings bolded to increase readability: Time 1 (COWAT: **0.90**; AF: **0.83**; S7: **0.82**; BDS: **0.87**; SDMT: **0.89**); Time 2 (COWAT: **0.85**; AF: **0.66**; S7: **0.80**; BDS: **0.83**; SDMT: **0.85**); Time 3 (COWAT: **0.86**; AF: **0.76**; S7: **0.75**; BDS: **0.85**; SDMT: **0.86**); Time 4 (COWAT: **0.85**; AF: **0.80**; S7: **0.81**; BDS: **0.79**; SDMT: **0.86**). Following this, PA and EFA were conducted, which similarly suggested a one-factor solution: Time 1 (COWAT: **0.89**; AF: **0.78**; S7: **0.76**; BDS: **0.84**; SDMT: **0.87**); Time 2 (COWAT: **0.80**; AF: **0.55**; S7: **0.73**; BDS: **0.80**; SDMT: **0.82**); Time 3 (COWAT: **0.82**; AF: **0.68**; S7: **0.68**; BDS: **0.82**; SDMT: **0.82**); Time 4 (COWAT: **0.82**; AF: **0.73**; S7: **0.75**; BDS: **0.73**; SDMT: **0.83**). It is also important to note that PCA, PA, and EFA, with a two-factor solution yielded factor loadings that did not meet the .5/.2 cutoff rule.

Confirmatory Factor Analysis

Table S3 shows that the one-factor EF solution had excellent model fit across all-time-points.

Table S3

*Confirmatory factor analysis of latent EF composite scores at all time-points*

|  |  | Factor loadings |  | Residual variances |
| --- | --- | --- | --- | --- |
| **Time 1** |  |  |  |  |
| Model fit indices: χ^2^(*df* = 5) = 0.132, *p* = 1.000, CFI = 1.000, RMSEA = .000 |  |  |  |  |
| Controlled word association test |  | 0.880^***^ |  | 0.225^***^ |
| Animal fluency |  | 0.771^***^ |  | 0.406^***^ |
| Serial 7s subtraction |  | 0.812^***^ |  | 0.341^***^ |
| Backward digit span |  | 0.824^***^ |  | 0.321^***^ |
| Symbol digit modalities test |  | 0.884^***^ |  | 0.219^***^ |
| Latent EF composite score |  | – |  | 1.000^***^ |
| **Time 2** |  |  |  |  |
| Model fit indices: χ^2^(*df* = 5) = 7.917, *p* = .161, CFI = 0.996, RMSEA = .048 |  |  |  |  |
| Controlled word association test |  | 0.784^***^ |  | 0.386^***^ |
| Animal fluency |  | 0.490^***^ |  | 0.760^***^ |
| Serial 7s subtraction |  | 0.675^***^ |  | 0.544^***^ |
| Backward digit span |  | 0.726^***^ |  | 0.473^***^ |
| Symbol digit modalities test |  | 0.844^***^ |  | 0.288^***^ |
| Latent EF composite score |  | – |  | 1.000^***^ |
| **Time 3** |  |  |  |  |
| Model fit indices: χ^2^(*df* = 5) = 3.738, *p* = .588, CFI = 1.000, RMSEA = .000 |  |  |  |  |
| Controlled word association test |  | 0.826^***^ |  | 0.318^***^ |
| Animal fluency |  | 0.700^***^ |  | 0.510^***^ |
| Serial 7s subtraction |  | 0.693^***^ |  | 0.520^***^ |
| Backward digit span |  | 0.792^***^ |  | 0.373^***^ |
| Symbol digit modalities test |  | 0.795^***^ |  | 0.368^***^ |
| Latent EF composite score |  | – |  | 1.000^***^ |
| **Time 4** |  |  |  |  |
| Model fit indices: χ^2^(*df* = 5) = 0.307, *p* = .998, CFI = 1.000, RMSEA = .000 |  |  |  |  |
| Controlled word association test |  | 0.785^***^ |  | 0.384^***^ |
| Animal fluency |  | 0.732^***^ |  | 0.464^***^ |
| Serial 7s subtraction |  | 0.746^***^ |  | 0.444^***^ |
| Backward digit span |  | 0.782^***^ |  | 0.388^***^ |
| Symbol digit modalities test |  | 0.804^***^ |  | 0.353^***^ |
| Latent EF composite score |  | – |  | 1.000^***^ |
|  |  |  |  |  |

Table S4

*Descriptive Statistics of Study Variables Across Time*

|  |  | *M* | (*SD*) | Lower CI | Upper CI |
| --- | --- | --- | --- | --- | --- |
| **Latent Scores** |  |  |  |  |  |
| NPI Depression Severity |  |  |  |  |  |
| Time 1 |  | 0.37 | (0.82) | 0.32 | 0.43 |
| Time 2 |  | 0.26 | (1.29) | 0.17 | 0.34 |
| Time 3 |  | 0.24 | (1.11) | 0.17 | 0.31 |
| Time 4 |  | 0.22 | (1.26) | 0.14 | 0.31 |
| NPI Anxiety Severity |  |  |  |  |  |
| Time 1 |  | 0.23 | (0.70) | 0.18 | 0.28 |
| Time 2 |  | 0.13 | (1.05) | 0.06 | 0.20 |
| Time 3 |  | 0.13 | (0.88) | 0.08 | 0.19 |
| Time 4 |  | 0.10 | (0.79) | 0.05 | 0.16 |
| Executive Functioning Composite | | |  |  |  |
| Time 1 |  | -0.00 | (1.08) | -0.07 | -0.00 |
| Time 2 |  | -0.13 | (1.43) | -0.22 | -0.03 |
| Time 3 |  | -0.80 | (1.55) | -0.91 | -0.70 |
| Time 4 |  | -0.97 | (1.67) | -1.09 | -0.86 |
| Controlled Oral Word Association Test | | |  |  |  |
| Time 1 |  | 18.40 | (14.69) | 17.41 | 19.38 |
| Time 2 |  | 18.70 | (20.63) | 17.31 | 20.08 |
| Time 3 |  | 17.11 | (20.39) | 15.74 | 18.48 |
| Time 4 |  | 17.02 | (22.41) | 15.52 | 18.52 |
| Animal Fluency |  |  |  |  |  |
| Time 1 |  | 10.84 | (6.20) | 10.42 | 11.25 |
| Time 2 |  | 11.07 | (7.87) | 10.54 | 11.59 |
| Time 3 |  | 10.40 | (9.45) | 9.77 | 11.03 |
| Time 4 |  | 10.13 | (10.53) | 9.43 | 10.84 |
| Serial 7s Subtraction |  |  |  |  |  |
| Time 1 |  | 2.09 | (1.08) | 1.96 | 2.23 |
| Time 2 |  | 2.09 | (1.43) | 1.92 | 2.27 |
| Time 3 |  | 1.96 | (1.55) | 1.77 | 2.15 |
| Time 4 |  | 1.80 | (1.67) | 1.57 | 2.02 |
| Backward Digit Span |  |  |  |  |  |
| Time 1 |  | 3.53 | (2.63) | 3.35 | 3.70 |
| Time 2 |  | 3.72 | (3.80) | 3.46 | 3.97 |
| Time 3 |  | 3.28 | (3.98) | 3.02 | 3.55 |
| Time 4 |  | 3.14 | (4.77) | 2.82 | 3.46 |
|  |  |  |  |  |  |

Table S4 (*continued*)

*Descriptive Statistics of Study Variables Across Time*

|  |  | *M* | (*SD*) | Lower CI | Upper CI |
| --- | --- | --- | --- | --- | --- |
| Symbol Digit Modalities Test |  |  |  |  |  |
| Time 1 |  | 15.28 | (15.42) | 14.25 | 16.32 |
| Time 2 |  | 14.23 | (19.02) | 12.95 | 15.50 |
| Time 3 |  | 12.27 | (19.28) | 10.98 | 13.56 |
| Time 4 |  | 10.42 | (23.96) | 8.82 | 12.03 |
|  |  |  |  |  |  |

*Notes.* CI = 95% confidence interval; NPI = neuropsychiatric inventory symptom severity.

Table S5

*Longitudinal Measurement Invariance of Study Variables*

|  |  | χ^2^ (*df*) | *p* | CFI | RMSEA [95% CI] |
| --- | --- | --- | --- | --- | --- |
| **Symptom Severity and EF**  Confirmatory factor analysis across time-points |  |  |  |  |  |
| Time 1 |  | 27.91 (62) | 1.000 | 1.000 | .000 [.000, .000] |
| Time 2 |  | 29.17 (62) | 1.000 | 1.000 | .000 [.000, .000] |
| Time 3 |  | 36.26 (62) | 1.000 | 1.000 | .000 [.000, .000] |
| Time 4 |  | 18.80 (62) | 1.000 | 1.000 | .000 [.000, .000] |
| Level of invariance across time-points |  |  |  |  |  |
| Configural (varying λ, τ, ε) |  | 112.15 (248) | 1.000 | 1.000 | .000 [.000, .000] |
| Metric (equal λ, varying τ, ε) |  | 185.42 (278) | 1.000 | 1.000 | .000 [.000, .000] |
| Scalar (equal λ, τ, varying ε) |  | 209.81 (308) | 1.000 | 1.000 | .000 [.000, .000] |
| Strict (equal λ, τ, ε) |  | 277.03 (347) | .998 | 1.000 | .000 [.000, .000] |
|  |  | Δχ^2^ (*df*) | *p* | ΔCFI | ΔRMSEA |
| Tests of measurement invariance across time-points |  |  |  |  |  |
| Metric vs. Configural |  | 73.27 (30) | < .001 | .000 | .000 |
| Scalar vs. Metric |  | 24.39 (30) | .754 | .000 | .000 |
| Strict vs. Scalar |  | 67.22 (39) | .003 | .000 | .000 |
|  |  |  |  |  |  |

*Note.* λ = item loading; τ = item intercept; ε = item error variance; CFI = confirmatory factor index; RMSEA = root mean square error of approximation.

Sensitivity Analysis of RI-CLPM and BLCS Models

As part of a sensitivity analysis, we reran all RI-CLPM and BLCS models in participants *without* significant cognitive impairment, clinical depression, physical, or cognitive disability (*n* = 611). Overall, the pattern of results remained similar to findings derived from the full sample. The RI-CLPM models showed acceptable fit for bivariate within-person level-to-future level relations between EF and depression severity (χ^2^(*df* = 25) = 39.703, *p* = .023, CFI = .987, RMSEA = .033) as well as between EF and anxiety severity (χ^2^(*df* = 25) = 131.956, *p* < .001, CFI = .912, RMSEA = .086). Likewise, acceptable model fit indices were observed for BLCS models testing the bivariate within-person change-to-future change associations between EF and depression severity (χ^2^(*df* = 25) = 58.440, *p* < .001, CFI = .972, RMSEA = .047) as well as between EF and anxiety severity (χ^2^(*df* = 23) = 66.831, *p* < .001, CFI = .964, RMSEA = .056).

Within persons, lower prior level of EF significantly predicted higher depression severity level at the next time-point (β = -0.082, 95% confidence interval [CI] [-0.135, -0.030], *d* = -0.292), and vice versa (β = -0.082, 95% CI [-0.135, -0.030], *d* = -0.292). Similarly, within persons, less increase in EF at one time-lag significantly forecasted more decrement in depression severity at the future adjacent time-lag (β = -0.698, 95% CI [-1.062, -0.334], *d* = -0.360), and vice versa (β = -0.698, 95% CI [-1.062, -0.334], *d* = -0.360). In addition, between persons, greater random intercept depression severity was significantly related to lower random intercept EF across all waves (β = -0.061, 95% CI [-0.100, -0.022], *d* = -0.293).

Also, cross-lagged relations between EF and anxiety severity were not significant, such that previous EF level did not predict anxiety severity at the next time-point within persons (β = -0.045, 95% CI [-0.110, 0.020], *d* = -0.131), and conversely (β = -0.045, 95% CI [-0.110, 0.020], *d* = -0.131). Likewise, coupling effects between EF and anxiety severity were not significant, such that within-person change in EF at one time-lag did not forecast change in anxiety severity at the next time-lag (β = 0.365, 95% CI [-0.171, 0.900], *d* = 0.128), and vice versa (β = 0.365, 95% CI [-0.171, 0.900], *d* = 0.128). However, between persons, stronger random intercept anxiety severity was significantly associated with lower random intercept EF across all time-points (β = -0.058, 95% CI [-0.089, -0.026], *d* = -0.348).

Figure S1

*Random-Intercept Cross-Lagged Panel Models Between EF and Anxiety Severity*

*Note.* ^**^ *p* < .01; ^***^*p* < .001.

ANX = anxiety severity; EF = executive functioning.

Figure S2

*Bivariate Dual Latent Change Score Models Between EF and Anxiety Severity*

**

*Note.* ^**^ *p* < .01; ^***^*p* < .001.

Δ = within-person change in construct from a time-lag to the next adjacent time-lag; ANX = anxiety severity; EF = executive functioning.

References

Lim, S., & Jahng, S. (2019). Determining the number of factors using parallel analysis and its recent variants. *Psychological Methods, 24*, 452-467. doi:10.1037/met0000230

Lubbe, D. (2019). Parallel analysis with categorical variables: Impact of category probability proportions on dimensionality assessment accuracy. *Psychological Methods, 24*, 339-351. doi:10.1037/met0000171

Matsunaga, M. (2010). How to factor-analyze your data right: do’s, don’ts, and how-to’s. *International Journal of Psychological Research, 3*, 97–110. doi:10.21500/20112084.854

Revelle, W. (2020). psych: Procedures for personality and psychological research, Northwestern University, Evanston, Illinois, USA, <https://CRAN.R-project.org/package=psych> Version = 2.0.9.

Rosellini, A. J., & Brown, T. A. (2021). Developing and validating clinical questionnaires. *Annual Review of Clinical Psychology*. doi:10.1146/annurev-clinpsy-081219-115343

Watkins, M. (2005). Determining parallel analysis criteria. *Journal of Modern Applied Statistical Methods, 5*, 344-346. doi:10.22237/jmasm/1162354020
